# Cholinergic Basal Forebrain Atrophy Accelerates Cognitive Decline via Cortical Thinning: The Moderating Role of Amyloid-β Pathology in Preclinical Alzheimer’s Disease

**DOI:** 10.21203/rs.3.rs-6220536/v1

**Published:** 2026-05-25

**Authors:** Wencai Ding, Cen Si, Lijuan Wang, Meiling Qiu, Zhongqiang Xu, Li Xu, Rui Bao, Xiaolei Tang, Juanyu Gong, Jinting Wu, Zhiding Shao, Tonghua Zhang, Fan Yang

**Affiliations:** The Second Affiliated Hospital of Wannan Medical College; The Second Affiliated Hospital of Wannan Medical College

## Abstract

Degeneration of the cholinergic basal forebrain (cBF) is a hallmark of early neurodegeneration in Alzheimer’s disease (AD). While cBF atrophy has been linked to cognitive decline, the extent to which cortical thinning mediates this association, particularly in preclinical AD, remains unclear. Furthermore, the influence of amyloid-β (Aβ) pathology on these relationships warrants further investigation. This study analyzed longitudinal MRI and PIB-PET data from 230 cognitively normal older adults in the Harvard Aging Brain Study, with a mean follow-up of six years. FreeSurfer was used to quantify cBF volume and cortical thickness, while cognitive performance was assessed using the Preclinical Alzheimer Cognitive Composite-5 (PACC5). Linear mixed-effects models evaluated longitudinal associations between cBF atrophy, cortical thinning, and cognitive decline. Mediation analyses assessed whether cortical thinning mediated the relationship between cBF degeneration and cognitive decline, and the moderating effect of Aβ burden was examined. Progressive cortical thinning in multiple cognition-related regions was significantly associated with longitudinal cBF atrophy. Mediation analysis revealed that cortical thinning accounted for approximately 44% of the association between cBF degeneration and cognitive decline. These effects were more pronounced in Aβ-positive individuals, suggesting a synergistic interaction between amyloid pathology and cholinergic degeneration. These findings indicate that cBF atrophy accelerates cognitive decline by driving cortical thinning, with Aβ pathology further exacerbating these effects. This study enhances understanding of the structural mechanisms underlying cognitive decline in preclinical AD and highlights potential early intervention targets.

## Introduction

Individuals with Alzheimer’s disease (AD) typically experience an extended period without cognitive symptoms before noticeable decline emerges[1]. Although definitions for preclinical AD vary, the widely accepted criterion includes normal cognitive function accompanied by at least one biomarker indicative of AD pathology, such as amyloid-beta (Aβ) deposits or tau pathology[2, 3]. However, amyloid plaque accumulation alone does not necessarily lead to Alzheimer’s disease, as it is also associated with the normal aging process. Therefore, relying solely on amyloid biomarkers is insufficient for fully characterizing preclinical AD[4, 5].

Brain atrophy, particularly in areas like the cholinergic basal forebrain (cBF), represents a hallmark pathological characteristic of Alzheimer’s disease[6]. Recent advancements in automated MRI techniques have facilitated the identification of in vivo neuroimaging biomarkers for basal forebrain atrophy[7, 8]. In individuals with mild cognitive impairment (MCI), atrophy of the basal forebrain cholinergic system has been associated with cortical thinning, especially in projection regions like the parahippocampal gyrus and cBF [9]. Research on Parkinson’s disease has demonstrated a correlation between basal forebrain atrophy, cortical hypometabolism, and cognitive decline[10]. In early stages of AD, basal forebrain atrophy combined with amyloid deposition significantly contributes to cognitive impairment[11, 12]. Therefore, imaging techniques such as MRI assessments of basal forebrain volume and cortical atrophy might identify early neurodegenerative changes, potentially aiding early diagnosis and interventions for preclinical AD.

Cortical thinning is also closely linked to cognitive decline. For instance, decreased cortical thickness in the left parahippocampal cortex has been associated with various cognitive functions[13]. Likewise, cortical thinning is observed in Parkinson’s disease patients at early stages, even before noticeable cognitive impairment develops, suggesting its potential role as an early indicator of cognitive decline[14]. Distinct patterns of cortical atrophy have also been observed across various MCI subtypes over three years. Memory-deficit MCI patients initially experience rapid atrophy in medial temporal areas, whereas naming/memory-deficit subtypes primarily show temporal lobe involvement, and mixed subtypes exhibit widespread cortical atrophy[15]. However, previous studies have been limited by methodological constraints, such as cross-sectional designs, small sample sizes, and single-center recruitment. These limitations have impeded a thorough investigation of the relationships among basal forebrain volume, cortical thinning, and cognitive function.

The current study addresses these limitations by utilizing a large multicenter cohort and longitudinal design to investigate these variables systematically over time. We hypothesize that cBF volume influences cognitive function through its effect on cortical thinning in specific brain regions. By exploring early-stage neurodegenerative changes, this study provides novel perspectives on the mechanisms that underlie cognitive decline in preclinical AD.

## Materials

### Study Design and Participants

This study enrolled 230 cognitively normal older adults from the Harvard Aging Brain Study (HABS), a prospective longitudinal cohort designed to investigate early neurodegenerative changes associated with preclinical Alzheimer’s disease. Conducted in collaboration between Massachusetts General Hospital and Brigham and Women’s Hospital in Boston, Massachusetts, the study aimed to enhance the understanding of early disease markers[16]. HABS integrates multimodal neuroimaging techniques to capture structural and functional brain alterations, including amyloid and tau pathology. These imaging assessments are complemented by detailed neuropsychological evaluations to track cognitive performance over time. The primary goal of these assessments is to identify biomarkers that could serve as early indicators of cognitive decline. The study received ethical approval from the Institutional Review Board (IRB) at Mass General Brigham, and all participants provided written informed consent. The research protocol complies with the ethical guidelines established in the Declaration of Helsinki.

### Neuropsychological Evaluation

Participants were identified as cognitively normal based on comprehensive neuropsychological evaluations. Inclusion criteria required the absence of clinical depression, defined as a Geriatric Depression Scale score of ≤ 10, with no history of active psychiatric disorders. Additionally, cognitive performance was evaluated using the Mini-Mental State Examination (MMSE), with a minimum score threshold of 25. Episodic memory was assessed using the Wechsler Logical Memory II delayed recall test, with participants required to score within 1.5 standard deviations of age- and education-adjusted norms. The Clinical Dementia Rating (CDR) scale was administered by trained clinicians blinded to biomarker status[16]. Global cognitive function was measured using the Preclinical Alzheimer Cognitive Composite-5 (PACC5), a composite index that integrates assessments across multiple cognitive domains, including episodic and semantic memory, executive function, and global cognition. The composite consists of the Mini-Mental State Examination (MMSE), Wechsler Memory Scale-Revised Logical Memory Delayed Recall (WMS-R LMDR), Digit-Symbol Coding Test (DSC), Free and Cued Selective Reminding Test-Free Recall (FCSRT96), and Category Fluency (CAT)[17]. The PACC5 serves as a sensitive tool for identifying early cognitive decline, beyond assessments of individual cognitive domains, and has been specifically designed to track subtle cognitive changes associated with Aβ pathology[17]. Participants underwent yearly cognitive assessments, incorporating evaluations of episodic memory, executive functioning, and global cognition, in conjunction with CDR scoring. Baseline cognitive assessments were conducted within a year of the initial MRI session.

### Imaging Acquisition and Processing

Structural MRI scans were acquired using a 3T scanner, following standardized imaging protocols to ensure consistency among participants. High-resolution T1-weighted images were obtained with a magnetization-prepared rapid gradient echo (MPRAGE) sequence. Imaging parameters included an isotropic voxel size of 1.0 mm^3^, an echo time (TE) of approximately 3.2 ms, a repetition time (TR) of ~ 2.2 s, and an inversion time (TI) of ~ 1.1 s. Parallel imaging techniques were employed to optimize signal quality and minimize scan duration. To assess amyloid-β (Aβ) burden, participants underwent positron emission tomography (PET) with Pittsburgh Compound B (^11^C-PIB)[18]. The cerebellar gray matter served as a reference region for computing standardized uptake value ratios (SUVR). Data preprocessing included motion correction and partial volume correction (PVC), utilizing validated imaging pipelines to enhance quantification accuracy.

### Basal Forebrain Volume

The cBF volume was used as an indicator of neuronal degeneration in cognitively normal older adults. Segmentation of the basal forebrain, was conducted on 3D T1-weighted MRI scans using the *ScLimbic* pipeline (https://surfer.nmr.mgh.harvard.edu/fswiki/ScLimbic)[19]. The total cBF volume was derived by summing the left and right basal forebrain volumes, as computed within each participant’s native T1-weighted structural MRI space. Following the execution of the *ScLimbic* pipeline, we conducted a visual inspection of the segmented images to detect any substantial inaccuracies in structure labeling. Furthermore, Additionally, the -*write_qa_stats* function in FreeSurfer’s *ScLimbic* pipeline was utilized to generate comprehensive quality assurance metrics.

### Cortical Thickness

Cortical thickness was measured using FreeSurfer software (version 6.0.0). The software calculates cortical thickness by determining the average distance between the gray-white matter boundary and the pial surface across the cortex[20, 21]. Image processing included several standard preprocessing steps: removing non-brain tissues, spatial normalization to a standard brain template, atlas registration, and parcellation using participant-specific brain templates. After these steps, we computed cortical thickness maps for each participant[22]. We visually inspected all individual cortical maps to ensure accuracy before including them in further analyses. To adjust for variations in head size, we adjusted cortical thickness measures using intracranial volume (ICV) estimated from FreeSurfer. Specifically, we applied the residual adjustment method, which corrects thickness measures based on variations in ICV [21, 23, 24]. The cortical thickness maps were aligned to the fsaverage standard template and processed with spatial smoothing using a Gaussian kernel with a 10 mm full-width at half-maximum (FWHM). Smoothing reduced variability between individuals and improved the reliability of statistical comparisons. This approach is consistent with best practices recommended for neuroimaging analyses.

### PIB-PET methods and quantification

**Figure 1:**
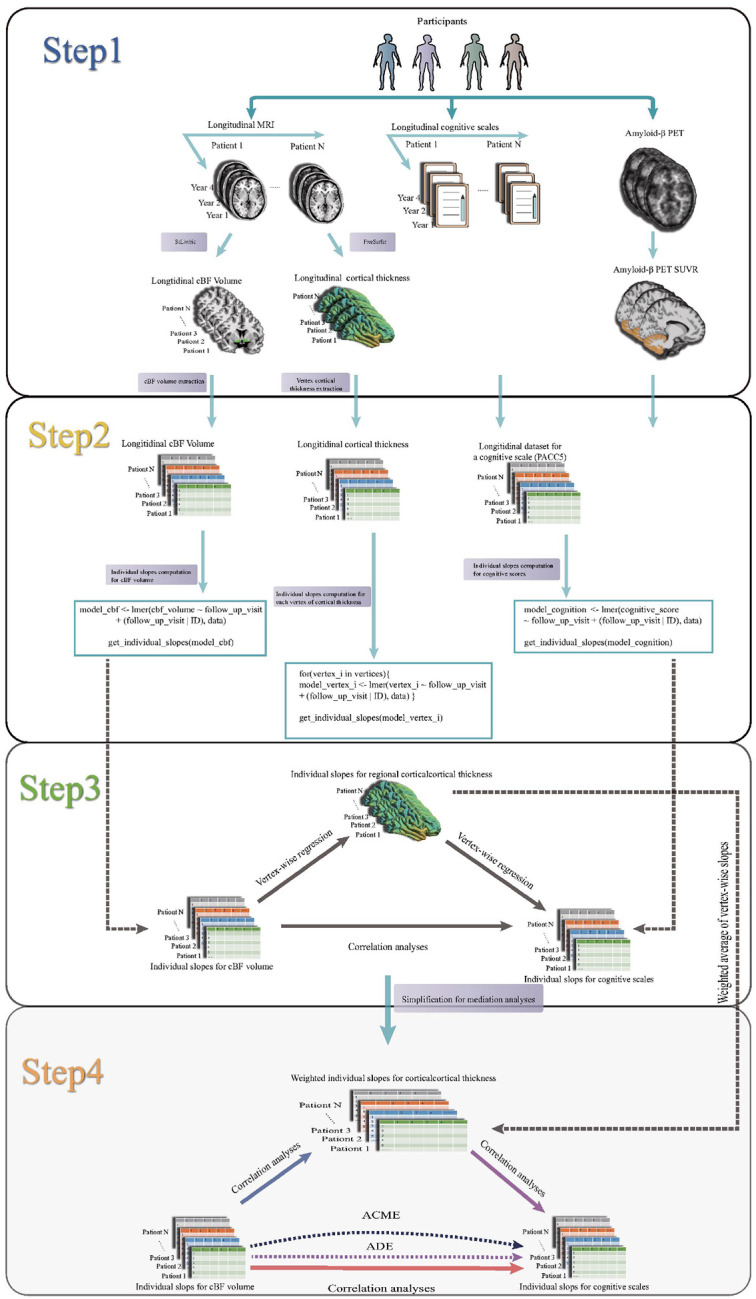
Study design flowchart outlining the complete analytical approach (details in METHODS).

Amyloid-β (Aβ) burden was assessed through positron emission tomography (PET) using Pittsburgh Compound B (^11^C-PIB). The scans were acquired at Massachusetts General Hospital with an ECAT EXACT HR+ scanner (Siemens, Erlangen, Germany)[16]. After an initial transmission scan, participants received an intravenous bolus injection of approximately 10–15 mCi ^11^C-PIB. A dynamic PET acquisition was conducted over a 60-minute period in three-dimensional mode, capturing 69 frames (12 × 15 s, 57 × 60 s) across 63 image planes with an axial field of view of 15.2 cm, trans-axial resolution of 5.6 mm, and slice thickness of 2.4 mm. To ensure alignment between PET images and anatomical structures, late-sum PIB-PET frames were co-registered to individual T1-weighted MRI scans using FreeSurfer’s *mri_coreg* tool. Amyloid deposition was measured using Logan graphical analysis, with the cerebellar gray matter serving as the reference region for calculating the distribution volume ratio (DVR). Non-partial volume corrected (non-PVC) DVR maps were generated and projected onto each participant’s cortical surface for regional analysis. Aβ burden was derived from a composite cortical region of interest, including the frontal, lateral temporal, parietal, and retrosplenial cortices, collectively known as PIB-FLR. Participants were categorized as amyloid-positive (Aβ+) or amyloid-negative (Aβ−) at baseline based on a PIB-FLR DVR threshold of 1.2, previously determined through Gaussian mixture modeling[25]. See Fig. 1 for an overview of the study design and analytical procedures (Fig. 1).

### Statistical analyses

Baseline differences in demographic, clinical, and cognitive characteristics between Aβ + and Aβ − groups were examined using independent-sample t-tests for continuous variables and chi-square tests for categorical variables. The Shapiro–Wilk test was used to evaluate normality, and log transformation was applied to non-normally distributed data when necessary.

To investigate longitudinal changes in cBF volume, cortical thickness, and cognitive performance, we utilized linear mixed-effects models over the follow-up period. These models incorporated participant-specific random intercepts and slopes to account for repeated measurements. Age, gender, and educational attainment were incorporated as covariates in the subsequent analyses. Before processing cortical thickness data, the ComBat harmonization method was applied to mitigate potential confounding effects related to site variability, age, sex, and educational background. ComBat is an empirical Bayesian approach designed to minimize variability from these confounding factors while preserving relevant biological signals[26]. We conducted vertex-wise analyses separately for each cortical location, estimating the rate of change (slope) over time.

We conducted mediation analyses to determine whether regional cortical thinning serves as an intermediary in the relationship between cBF degeneration and cognitive decline in cognitively normal older adults. First, we examined the relationship between longitudinal cBF volume reduction and cognitive performance by computing individual slopes of change. We repeated this analysis for the relationship between cortical thinning and cognitive decline. We then identified cortical regions significantly associated with cognitive decline, weighting each region’s cortical thinning slope according to its strength of association with cognition.

We conducted additional mediation analyses to further explore the relationships among basal forebrain atrophy, cortical thinning, and cognitive decline. First, we tested whether longitudinal reductions in basal forebrain volume were associated with cognitive decline. Next, we examined the relationship between cortical thinning in basal forebrain-related regions and cognitive performance using vertex-wise regression analyses. Rather than calculating a simple average cortical thinning across all significant regions, we weighted the cortical thinning slopes according to the strength of their association with cognitive decline. We conducted mediation analyses to assess whether cortical thinning acts as an intermediary in the association between cBF degeneration and cognitive decline. Specifically, we measured the average direct effect (ADE), representing the direct impact of basal forebrain degeneration on cognitive decline, and the average causal mediation effect (ACME), which captures the indirect effect mediated by cortical thinning. We used nonparametric bootstrapping with 10,000 resamples to determine the statistical significance of these effects and to estimate the proportion of mediation.

We conducted additional analyses to evaluate whether cortical thinning independently contributes to cognitive decline, separate from cBF degeneration. To investigate this relationship, we conducted vertex-wise regression analyses to evaluate the relationship between cortical thinning and longitudinal changes in PACC5 scores. We calculated the average cortical thinning effect across all significant vertices, both before and after adjusting for longitudinal changes in cBF volume. The primary measure of cognitive decline in these analyses was the slope of the PACC5 score change. Vertex-wise analyses were adjusted for multiple comparisons using permutation testing with 10,000 iterations, employing a two-tailed cluster-forming threshold of *P* < 0.05. See the flowchart in Fig. 1 for the study design and analytical approach (Fig. 1). All statistical analyses were performed using R version 4.4.0. Linear mixed-effects models and mediation models were implemented utilizing the lme4 package and the mediation package, respectively.

## Results

### Demographic Characteristics

Table 1 summarizes the demographic, clinical, and cognitive characteristics of study participants. A total of 230 individuals were included, with 61 (26.5%) classified as Aβ + and 169 (73.5%) as Aβ −. The mean age of the Aβ + group was 74.7 years (SD = 5.37), and 55.7% were female. The frequency of the APOE-ε4 allele was markedly greater in the Aβ + group (59.0%) than in the Aβ − group (16.6%, *P* < 0.001). Additionally, amyloid burden, measured by PIB-FLR DVR, was notably elevated in the Aβ + group (mean = 1.45, SD = 0.167) relative to the Aβ − group (mean = 1.08, SD = 0.0511, *P* < 0.001). Participants in the Aβ + group underwent MRI assessments for an average of 4.14 years (SD = 1.45), with approximately 2.69 follow-up scans (SD = 0.672). While PACC5 scores were marginally lower in the Aβ + group (mean = −0.0018, SD = 0.590) than in the Aβ − group, this difference did not reach statistical significance (*P* = 0.42). The Aβ − participants had a mean age of 72.9 years (SD = 6.32) and a slightly higher proportion of female participants (59.8%). Their PIB-FLR DVR values were significantly lower than those of the Aβ + group (mean = 1.08, SD = 0.0511, P < 0.001).

For the Aβ − group, the average MRI follow-up duration was 4.48 years (SD = 1.43), with an average of 2.80 MRI scans per participant (SD = 0.684). Mini-Mental State Examination (MMSE) scores were comparable between the two groups, with no significant difference (Aβ−: mean = 29.1, SD = 1.05; Aβ+: mean = 28.8, SD = 1.04, *P* = 0.061). Similarly, PACC5 scores did not significantly differ (Aβ−: mean = 0.0715, SD = 0.649; *P* = 0.42). Both groups underwent a similar number of cognitive assessments, averaging 5.77 assessments (SD = 0.500) in the Aβ − group.

### Longitudinal Trajectories of cBF Atrophy, Cortical Thinning, and Cognitive Decline

At baseline, cBF volume and ICV showed no significant differences between Aβ + and Aβ − groups (Supplementary Fig. 1). However, all participants demonstrated significant declines in cBF volume over time (β = −4.49; 95% CI: [− 5.43, − 3.55]; T = − 7.10; *P* < 0.001). When stratified by amyloid status, both Aβ + and Aβ − groups exhibited significant cBF volume reductions, with a more pronounced decline observed in Aβ + individuals (β = −5.93; 95% CI: [− 8.21, − 3.66]; T = − 5.15; *P* < 0.001), whereas the Aβ − group demonstrated a comparatively smaller decrease (β = −3.89; 95% CI: [− 4.96, − 2.81]; T = − 7.10; *P* < 0.001).(Supplementary Table 1). Similarly, significant progressive cortical thinning occurred across extensive brain regions during follow-up. Regions exhibiting significant cortical thinning over the follow-up period included the entorhinal cortex, fusiform gyrus, parahippocampal gyrus, inferior and middle temporal gyri, inferior parietal lobule, insula, isthmus cingulate, precuneus, and occipital cortex bilaterally (Supplementary Fig. 2). Cognitive performance, assessed by PACC5 scores, significantly declined over time across all participants (β = −0.03; 95% CI: [− 0.04, − 0.01]; T = − 2.90; *P* = 0.004). When analyzed separately by amyloid status, the Aβ + group showed a pronounced cognitive decline (β = −0.11; 95% CI: [− 0.15, − 0.06]; T = − 4.50; *P* < 0.001). In contrast, no significant cognitive decline was observed in the Aβ − group (β = 0.003; 95% CI: [− 0.01, 0.02]; T = 0.48; *P* = 0.635) (Supplementary Table 2).

### Longitudinal cBF Degeneration Associates with Parallel Cortical Thinning

**Figure 2:**
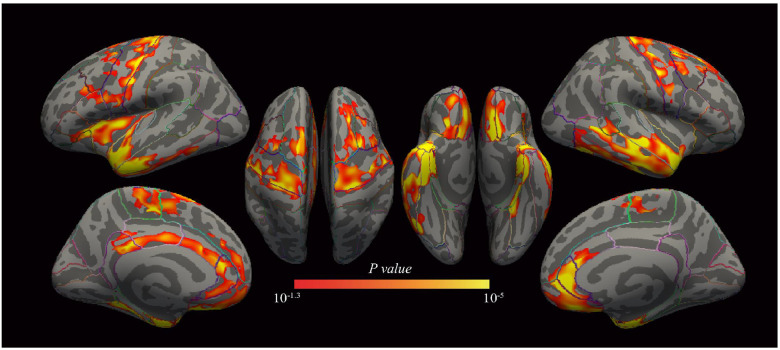
Association between longitudinal cBF volume atrophy and cortical thinning in all participants. The figure illustrates the regional effects of a regression analysis examining the relationship between the rate of change in cBF volume and the rate of change in vertex cortical thickness within a normal aging population. After adjusting for age, sex, and years of education, and applying permutation test correction, significant associations between cBF atrophy and cortical thinning were identified. Only clusters that met family-wise error correction at *P* < 0.05 are shown.

We employed linear mixed-effects models to investigate the relationship between longitudinal cBF volume decline and cortical thinning. The models incorporated time as a fixed effect and participant as a random effect, adjusting for age, sex, and education using ComBat harmonization. To control for multiple comparisons, permutation testing with threshold-free cluster enhancement (TFCE) was applied. Significant associations were observed between cBF volume reduction and cortical thinning across various cortical areas (Fig. 2). Strong correlations were identified in bilateral temporal regions, including the inferior, middle, and superior temporal gyri, temporal poles, entorhinal cortex, and parahippocampal gyri. In the frontal lobes, notable associations were detected in the medial orbitofrontal cortex, anterior cingulate cortex, and precentral gyrus. The bilateral insular cortex also demonstrated significant thinning associated with declining cBF volume. Specifically, in the left hemisphere, significant effects appeared in the temporal pole, insula, precentral gyrus, medial orbitofrontal cortex, and cingulate cortex. In the right hemisphere, significant associations occurred primarily in the superior frontal gyrus, anterior cingulate cortex, and entorhinal cortex (Fig. 2).

### Cortical Thinning as a Mediator of cBF Degeneration and Cognitive Decline

**Figure 3:**
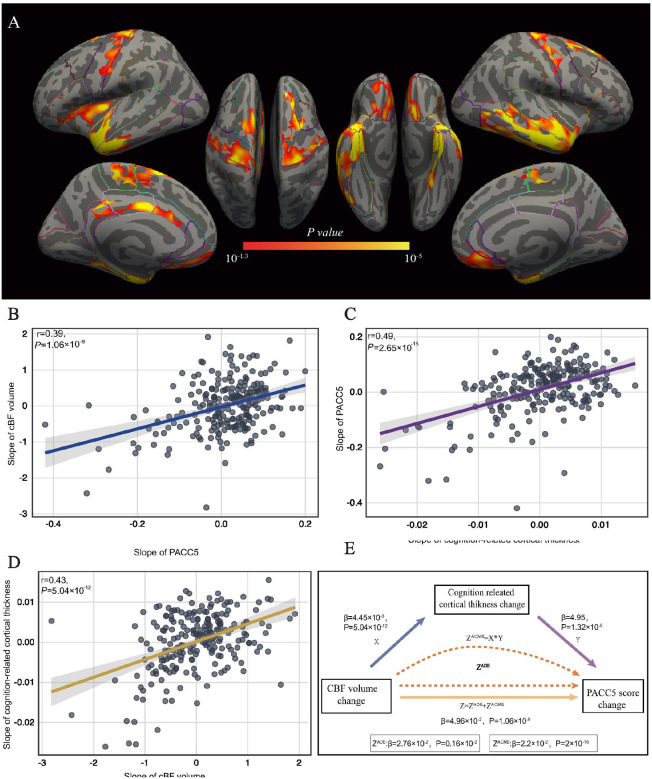
Mediation analysis of associations between cholinergic basal forebrain (cBF) degeneration, cortical thinning, and cognitive decline. **A.** Cortical clusters showing significant correlations between vertex-wise slopes of cortical thickness change and the slope of change in Preclinical Alzheimer Cognitive Composite-5 (PACC5) score. Vertex-wise regression analyses were restricted to cortical areas showing significant associations with cBF degeneration (as shown in Fig. 2) and corrected for multiple comparisons using permutation testing. Only clusters that met family-wise error correction at *P*< 0.05 are shown. **B.** Individual slopes of change in PACC5 score (x-axis) are plotted against individual slopes of change in cBF volume (y-axis). The blue line represents the linear trend. **C.** Average individual slopes of thickness change in cognition-related cortical areas (x-axis) are plotted against individual slopes of change in PACC5 score (y-axis). The purple line represents the linear trend. **D.** Individual slopes of change in cBF volume (x-axis) are plotted against average individual slopes of thickness change in cognition-related cortical areas (y-axis). The yellow line represents the linear trend. **E.** Path diagram of the causal mediation model, where the effect of cBF degeneration on cognitive decline is plotted in path Z (yellow arrow), the effect of cBF degeneration on cortical thinning is plotted in path X (blue arrow), and the effect of cortical thinning on cognitive decline is plotted in path Y (purple arrow). Betas [95% confidence intervals] and P values of regression analyses are indicated for each association. The total effect of path Z is composed of the sum of the average direct effect (ZADE) and the average causal mediation effect (ZACME) (purple arrows).

We further investigated whether cortical thinning acts as an intermediary in the association between cBF degeneration and cognitive decline in cognitively normal older adults. Cortical thinning in cBF-associated regions significantly correlated with cognitive decline. Specifically, cognitive decline was related to cortical thinning in several brain regions (Fig. 3A). In the left hemisphere, cortical thinning was observed in the temporal lobe, entorhinal cortex, parahippocampal gyrus, insular cortex, medial orbitofrontal cortex, cingulate cortex, and precentral gyrus. In the right hemisphere, it primarily affected the temporal lobe, entorhinal cortex, superior frontal gyrus, medial orbitofrontal cortex, anterior cingulate cortex, and precentral gyrus. Longitudinal cBF atrophy showed a significant correlation with declines in PACC5 scores (r = 0.39, *P* = 1.06 × 10^−9^) (Fig. 3B). Mediation analysis revealed that cortical thinning accounted for approximately 44.34% of the total effect of cBF degeneration on cognitive decline (β = 0.0496; 95% CI [0.03, 0.07]; *P* = 1.06 × 10^−9^). Both the direct effect of cBF degeneration (ADE: β = 0.0276; 95% CI [0.01, 0.04]; *P* = 0.0016) and the indirect effect mediated by cortical thinning (ACME: β = 0.0220; *P* < 2 × 10^−16^) were statistically significant (Fig. 3C–E).

### Effects of Amyloid-β Pathology on the Observed Associations

**Figure 4:**
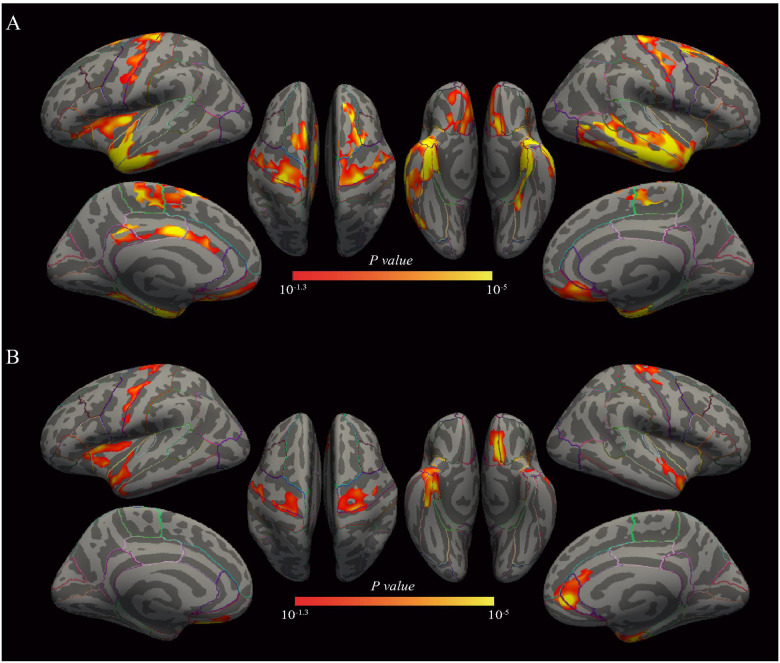
Associations between longitudinal cholinergic basal forebrain (cBF) degeneration and regional cortical thinning in Aβ+ and Aβ− groups. The figure shows regional effects of regression analyses of the slope of change in cBF volume on vertex-wise slopes of change in cortical thickness, separated by Aβ+ and Aβ− groups. After adjusting for age, sex, and years of education, and applying permutation test correction, significant associations between cBF atrophy and cortical thinning were identified. Only clusters that met family-wise error correction at *P* < 0.05 are shown.

Next, we examined how Aβ pathology influences the relationships among cBF atrophy, cortical thinning, and cognitive decline. Interestingly, longitudinal cBF atrophy did not significantly differ between Aβ + and amyloid-negative Aβ − participants (Supplementary Fig. 3). However, significant associations between cBF degeneration and cortical thinning were influenced by amyloid-β status. Specifically, regional cortical thinning associated with cBF degeneration was more pronounced in Aβ + participants compared to those without amyloid pathology. The regions showing these amyloid-dependent effects included the medial orbitofrontal cortex, temporal pole, entorhinal cortex, and parahippocampal cortex (Fig. 4A–B).

### Effects of Amyloid-β Pathology on the Mediation of Cortical Thinning

We further investigated how Aβ pathology influences cortical thinning as a mediator between cBF degeneration and cognitive decline. In the Aβ + group, longitudinal cBF degeneration significantly correlated with declines in cognitive performance, measured by the PACC5 (r = 0.26, *P* = 5.59 × 10^−4^; Supplementary Fig. 4B). Mediation analyses revealed that cortical thinning served as a partial mediator in the association between cBF degeneration and cognitive decline (total effect: β = 0.0902; 95% CI [0.06, 0.12]; *P* = 3.77 × 10^−16^). Both the direct effect (ADE: β = 0.0604; *P* = 0.017) and indirect effect through cortical thinning (ACME: β = 0.0299; *P* = 0.044) were statistically significant, with cortical thinning mediating approximately 33.16% of the total effect (Supplementary Fig. 4C–E). Similarly, in the Aβ − group, longitudinal cBF degeneration strongly correlated with cognitive decline (r = 0.39, *P* = 1.06 × 10^−9^; Supplementary Fig. 5B). Mediation analyses indicated that cortical thinning played a mediating role in the link between cBF atrophy and cognitive impairment among Aβ − participants (total effect: β = 0.0248; 95% CI [0.01, 0.04]; *P* = 0.0008). Again, both direct (ADE: β = 0.0148; *P* = 0.035) and indirect effects (ACME: β = 0.0100; *P* = 0.0004) were significant, with cortical thinning accounting for approximately 40.39% of the total effect (Supplementary Fig. 5C–E).

## Discussion

This study examined the longitudinal relationships among cBF degeneration, cortical thinning, and cognitive decline in cognitively normal older adults at the preclinical stage of AD. Additionally, we investigated whether Aβ pathology, as measured by PET imaging, modulates these associations. Our findings indicate that progressive cBF degeneration is significantly associated with cortical thinning in regions essential for cognitive function. Furthermore, cortical thinning serves as an intermediary factor linking cBF degeneration to cognitive decline. Notably, the association between cBF degeneration and cortical thinning was stronger in Aβ + individuals compared to Aβ- individuals, suggesting that Aβ pathology may exacerbate neurodegenerative processes linked to cholinergic system dysfunction.

In this study, we observed that even during the preclinical stage of AD—before the onset of cognitive decline—cortical thinning in regions associated with cognitive function closely corresponds to areas exhibiting cBF atrophy. This finding suggests a potential interrelationship between cortical thinning, cBF degeneration, and cognitive impairment. The loss of cholinergic neurons is a well-established neuropathological feature of AD-related dementia[27–29], and recent in vivo MRI morphometry studies have further explored the cognitive consequences of cBF degeneration in the preclinical phases of AD[30]. These studies demonstrate that reduced cBF volume, as measured by MRI, is strongly linked to cognitive impairment in AD patients. Moreover, our findings highlight the critical role of cBF atrophy in mediating the relationship between Aβ accumulation and early cognitive decline. Consistent with previous cross-sectional analyses, our longitudinal MRI data reveal that progressive cBF degeneration is accompanied by both global and domain-specific cognitive decline.

To further investigate this relationship, a longitudinal mediation analysis was performed to investigate the impact of cBF atrophy, cortical thinning in relevant regions, and cognitive decline. Our findings indicate that both cortical thinning and cBF atrophy are significantly associated with cognitive decline. Additionally, reduced cBF volume at baseline predicted future cognitive decline in individuals who were cognitively intact at study entry. Consistent with our results, previous MRI morphometry studies have reported associations between cortical atrophy and cognitive impairment across the AD continuum, particularly in the frontotemporal, insular, and posterior cortical regions[31, 32]. Temporal lobe atrophy, in particular, has been strongly linked to cognitive decline in AD, with studies demonstrating significant bilateral atrophy in the medial temporal lobes across various AD subtypes[33]. Recent evidence further suggests that basal forebrain degeneration not only precedes but also predicts the subsequent spread of AD pathology to cortical regions[34, 35]. Additionally, neuroimaging research has identified in vivo links between cBF degeneration and atrophy in its extensively innervated cortical projection areas[36]. Building upon these insights, our study is the first to establish a similar in vivo association between progressive cBF degeneration and simultaneous cortical thinning in preclinical AD. Furthermore, we observed that this association is linked to cognitive decline even in the earliest stages of the disease. However, similar findings have been reported in Parkinson’s disease, indicating that cBF degeneration and cortical atrophy may be interrelated processes contributing to cognitive decline in neurodegenerative disorders beyond AD[37, 38]. While our results suggest that cBF degeneration influences cognitive decline primarily through its effect on cortical thinning, this does not exclude the possibility that cortical thinning independently contributes to cognitive deterioration in preclinical AD. Our supplementary analyses assessing the independent effects of cortical atrophy on cognitive decline support the notion that cortical thinning is the most immediate structural correlate of cognitive impairment. Furthermore, aspects of this process appear to be linked to the degeneration of cortically projecting cBF neurons, reinforcing its mediating role in cognitive decline.

Our findings on the cortical mediation of cognitive decline associated with cBF degeneration are consistent with similar results reported in Parkinson’s disease[37, 39]. Research using selective cBF lesions in animal models has shown that these lesions lead to neuronal dysfunction in the corresponding cortical regions[40]. The severity of cognitive deficits appears to correlate with the extent of cortical dysfunction. One plausible mechanism is that the loss of cholinergic innervation exacerbates pathological processes in affected cortical areas, potentially due to impaired clearance mechanisms. Recent studies emphasize the essential role of cholinergic innervation in maintaining metabolic and housekeeping functions in cortical neurons[41]. Furthermore, experimental AD models demonstrate that selective cBF lesions accelerate cortical Aβ accumulation[42, 43]. However, the directionality of these associations remains uncertain. An alternative explanation is that cortical atrophy induces retrograde degeneration of the cBF due to a lack of trophic support. Cholinergic neurons, with their extensive axonal projections to cortical and subcortical regions, rely heavily on trophic factors for maintenance and molecular transport. Disruptions in these processes may result from primary neurodegeneration in cortical target areas, further contributing to cBF atrophy.

To determine whether cBF degeneration is primarily driven by AD pathology, we conducted an explicit assessment using Aβ PET as a biomarker of AD pathology. Our findings indicate that cBF volume reduction correlates with cortical thinning, with more pronounced thinning observed in the Aβ + group compared to the Aβ − group. The relationship between progressive cBF degeneration and cortical thinning was partially influenced by Aβ status, indicating that these processes are integral to AD pathophysiology. Previous cross-sectional studies have shown that individuals with mild cognitive impairment exhibit reduced cBF volume, which is associated with cognitive deficits[30]. This reduction is strongly correlated with decreased metabolic activity across widespread cortical networks, particularly in the prefrontal cortex, medial temporal lobe, and temporoparietal junctio [44]. The loss of cholinergic fibers is believed to contribute to cortical thinning in these regions by impairing synaptic plasticity and neuronal communication, thereby accelerating cognitive decline[45]. Additionally, disruptions in acetylcholine’s role in regulating the blood-brain barrier may lead to abnormal metabolite transport between interstitial and cerebrospinal fluids. This dysfunction could impair Aβ clearance, exacerbating AD pathology by promoting Aβ accumulation, neurodegeneration, cBF atrophy, and cortical thinning[46]. These findings suggest that cholinergic deficits in the basal forebrain disrupt both neuronal function and cortical structure, potentially explaining the link between cBF volume loss and cortical thinning observed in our study.

This study has several limitations that warrant consideration. First, while we investigated the mediating role of cortical thinning in the relationship between cBF atrophy and cognitive decline in AD, our findings do not establish causality. The precise pathological mechanisms linking these factors remain uncertain. Second, the mediating effect of cortical thinning was relatively modest, suggesting that additional factors contribute to the relationship between cBF degeneration and cognitive function. Future research should explore the role of neuroinflammation, synaptic dysfunction, and other biomarkers to gain a more comprehensive understanding of this complex interplay. Finally, this study focused on individuals in the preclinical phase of AD over a relatively short follow-up period. Longer longitudinal studies are needed to determine whether these relationships evolve as individuals progress to clinically diagnosed AD. Additionally, future research should investigate whether early interventions targeting cBF atrophy, cortical thinning, or cholinergic dysfunction could help delay or prevent AD onset.

In conclusion, Our findings highlight the critical role of cholinergic system degeneration in the onset of cognitive deficits during the preclinical stage of AD. Moreover, they provide new insights into the relationship between cBF atrophy, regional cortical thinning, and Aβ pathology—two well-established biomarkers of cognitive decline in AD. These associations have important implications for biomarker-based disease prognosis and the stratification of participants in clinical trials. Additionally, our results suggest that cholinergic interventions may warrant re-evaluation, particularly in the predementia stages of AD, as targeting cholinergic dysfunction early in the disease process could offer therapeutic benefits.

## Supplementary Material

Table 1 is available in the Supplementary Files section.

This is a list of supplementary files associated with this preprint. Click to download.

- SupplementaryTable2.docx
- SupplementaryFig.3.pdf
- SupplementaryTable1.docx
- SupplementaryFig.4.pdf
- SupplementaryFig.2.pdf
- SupplementaryFig.1.pdf
- SupplementaryFig.5.pdf
- Table.docx
- SUPPLEMENTALMATERIAL.docx
